# 4D Millimeter-Wave Radar Point Cloud Sensing for Face Guard Detection and Cutting-Interference Warning in Fully Mechanized Coal Mining Faces

**DOI:** 10.3390/s26185919

**Published:** 2026-09-19

**Authors:** Yihui Zhao, Zhongbin Wang, Mo Chen, Dong Wei

**Affiliations:** 1 School of Mechatronic Engineering, China University of Mining and Technology, No.1 Daxue Road, Xuzhou 221116, China; xamjzhaoyihui@163.com (Y.Z.); chenmo_cumt@126.com (M.C.); 2 Xi’an Coal Mining Machinery Co., Ltd., No.7 Jingpu Road, Gaoling District, Xi’an 710200, China

**Keywords:** 4D millimeter-wave radar, radar point cloud, PointNet++, semantic segmentation, hydraulic support, face guard, cutting interference, coal mining safety, 3D sensing

## Abstract

Spatial interference among the shearer cutting drum, ranging arm, and hydraulic-support face guard is a practical safety risk in fully mechanized coal mining faces, where dust, water mist, weak illumination, and metal multipath reflections limit vision- and LiDAR-based perception. This study develops a 4D millimeter-wave radar point cloud sensing method for face guard segmentation and cutting-interference warning. Radar point clouds were extracted from ROS bag data, converted to frame-level point cloud files, manually labeled, and processed using a joint passthrough-radius filtering strategy. A PointNet++ semantic segmentation network was modified with radar feature augmentation and class-aware neighborhood sampling to identify sparse face guard points from background structures. The segmented face guard points were fitted by an axis-aligned 3D bounding box, and the minimum distance from shearer dangerous regions to the box was used to classify safe, warning, and dangerous states. On a 2160-frame labeled dataset, the final segmentation model reached 99.126% precision, 97.477% recall, and 96.608% IoU at epoch 100. Static pose, dust-condition, and dynamic sequence tests further verified that the proposed sensing pipeline can provide continuous face guard localization and distance-based interference monitoring under representative experimental conditions.

## 1. Introduction

Fully mechanized coal mining faces are narrow, dusty, and mechanically crowded workspaces in which the coal shearer, scraper conveyor, and hydraulic supports operate in a tightly coupled sequence. During cutting, the rotating shearer drum and ranging arm pass close to the hydraulic-support face guard. If the face guard is not retracted or positioned with sufficient clearance, cutting interference can damage picks, drums, guard plates, and support structures and can interrupt production or compromise local support stability [1]. Continuous perception of the relative spatial relationship between the shearer cutting mechanism and the face guard is therefore important for active safety monitoring.

Reliable sensing in this environment is difficult. Visible-light cameras depend on illumination and unobstructed imaging, while underground coal dust, water mist, vibration, and low light can severely degrade image quality. LiDAR provides accurate 3D geometry, but near-infrared laser beams can be attenuated or scattered by suspended dust and water droplets, creating dense noise and missing surfaces behind dust clouds [2,3,4]. Millimeter-wave radar has a longer operating wavelength and is less sensitive to illumination and fine suspended particles. Compared with conventional 3D radar that mainly provides range, radial velocity, and azimuth, emerging 4D millimeter-wave radar also estimates elevation, enabling a sparse but useful three-dimensional point cloud for near-field equipment monitoring [5,6].

The direct use of 4D radar point clouds in underground machinery monitoring remains challenging. First, the metal-rich structure of a fully mechanized face can create multipath ghosts and isolated reflections [7,8]. Second, a single 4D radar frame is sparse and may not describe the complete geometry of a face guard. Third, the target points form a minority class relative to background points from the coal wall, drum, ranging arm, support, and experimental fixtures. Fourth, a collision-warning output requires more than object recognition: the target point cloud must be converted into a spatial occupancy model and compared with the dangerous regions of the shearer mechanism.

Prior work on point cloud processing provides useful components but does not directly solve this ultra-near-field mining scenario. Classical clustering methods such as Euclidean clustering and density-based spatial clustering of applications with noise (DBSCAN) can separate point clusters under suitable density and distance settings [9,10,11]. However, their parameter sensitivity is problematic when target density changes with radar angle, distance, and multipath. Deep point cloud networks, including PointNet and PointNet++, directly process unordered point sets and learn discriminative local features [12,13]. Radar-oriented semantic segmentation and 4D radar enhancement methods have also been explored in autonomous driving and underground perception [14,15,16,17,18]. Nevertheless, a practical pipeline that combines 4D radar preprocessing, face guard point segmentation, bounding-box generation, and shearer interference warning remains underdeveloped.

The main contributions are as follows:

1. A 4D millimeter-wave radar point cloud dataset construction workflow is established for face guard detection, including ROS bag parsing, PointCloud2 extraction, point cloud file export, CloudCompare labeling, and frame-level organization.
2. A radar point cloud preprocessing and semantic segmentation pipeline is proposed. Passthrough filtering and radius filtering suppress irrelevant spatial regions and isolated noise; a PointNet++ model with radar feature augmentation and class-dependent anchor-neighborhood sampling improves the segmentation of sparse minority target points.
3. A distance-based cutting-interference warning method is developed by converting segmented face guard points into an axis-aligned 3D bounding box and computing the minimum distance from shearer dangerous regions to this box.
4. Static pose tests, normal/dust condition tests, and dynamic sequence tests verify that the proposed 4D radar sensing pipeline can continuously identify the face guard and support interference-state output in representative experimental scenarios.

## 2. Materials and Methods

### 2.1. Experimental Scenario and Hardware Configuration

**Figure 1:**
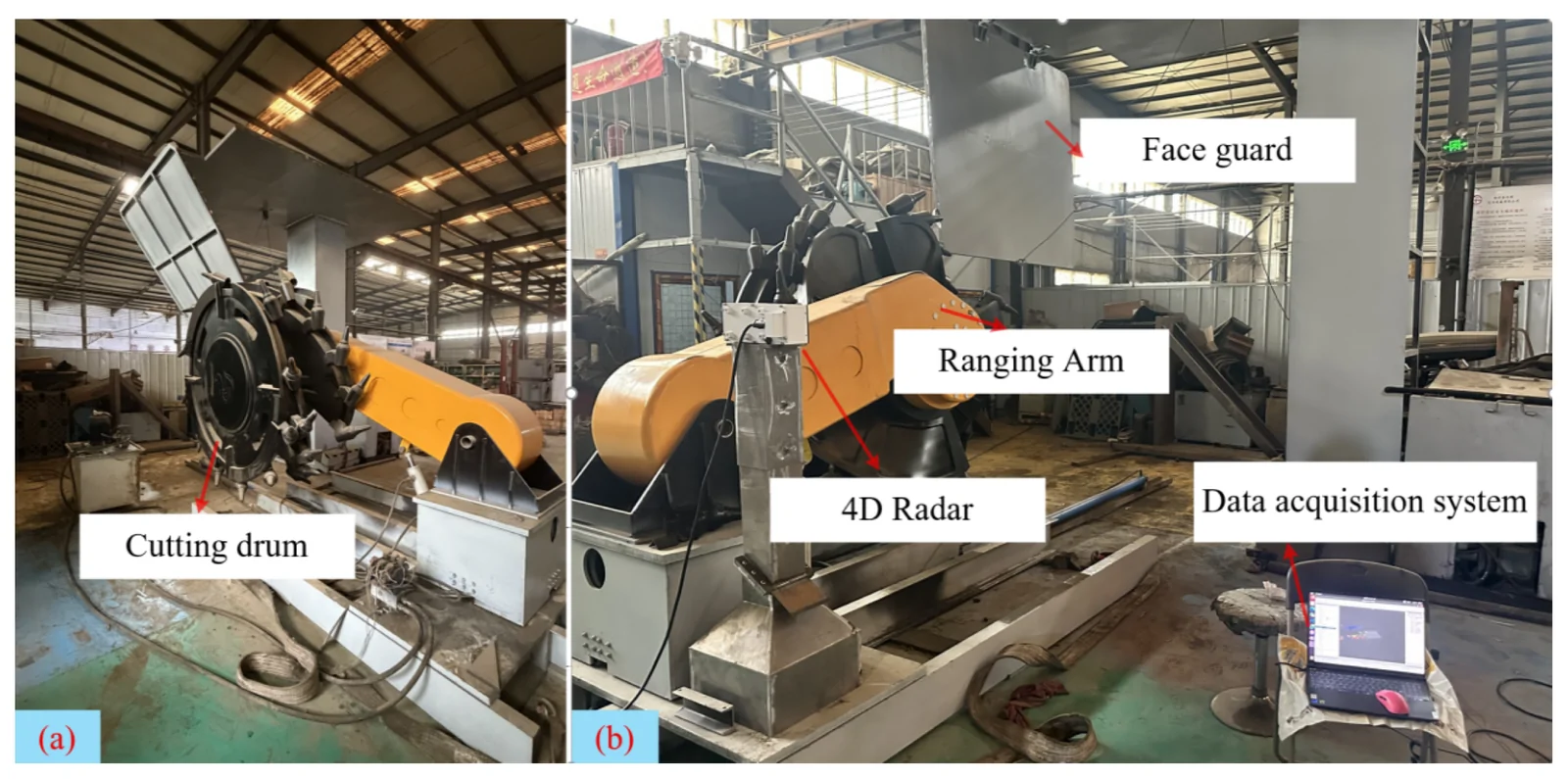
Experimental platform for 4D millimeter-wave radar point cloud acquisition and cutting-interference validation. The platform includes a simulated cutting drum and ranging arm, a hydraulic-support face guard, a 4D radar, and a data acquisition computer. (**a**) Cutting drum, (**b**) Simulate interference structures and perception systems.

The study focuses on the spatial relationship between the shearer cutting mechanism and the hydraulic-support face guard. The face guard is mounted near the coal-wall side of the support and can extend or retract to protect the coal wall. When the shearer approaches, the face guard must leave enough space for the drum and ranging arm. The experimental platform (Figure 1) was built to reproduce this local spatial relationship and to collect 4D radar point clouds under different face guard poses and environmental conditions.

**Figure 2:**
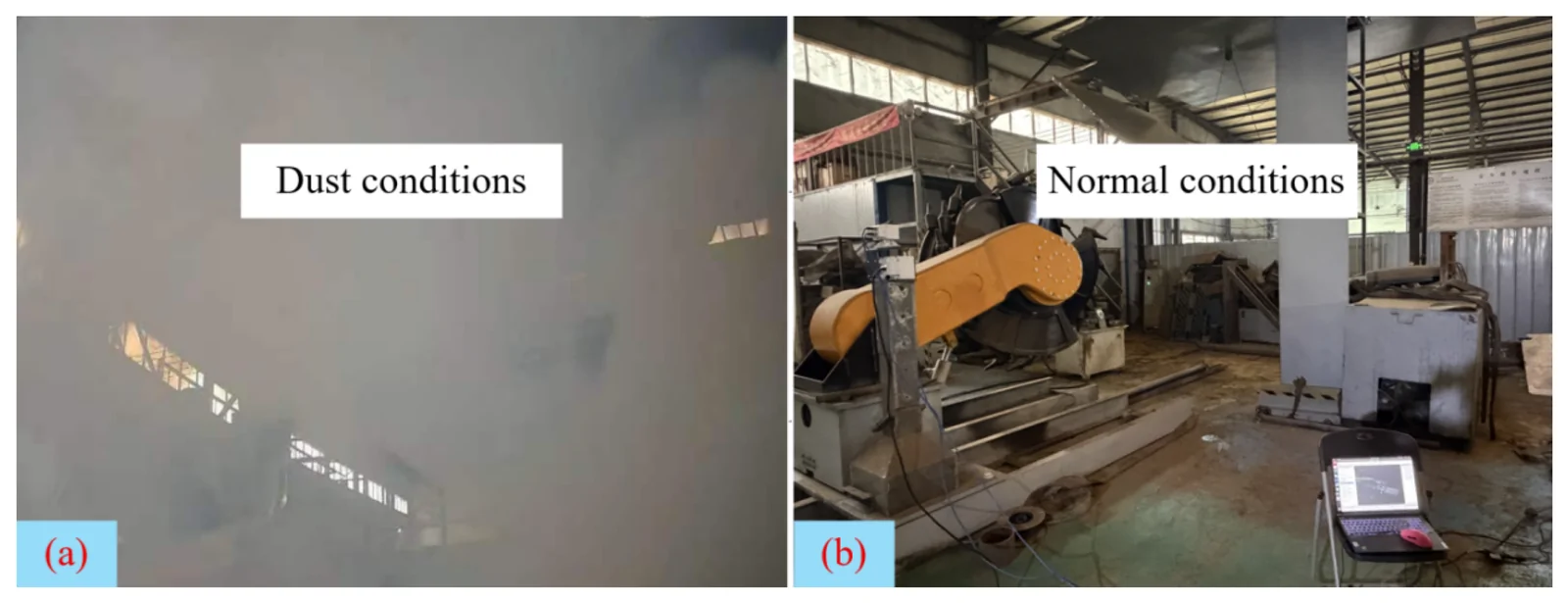
Experimental visual comparison between dust and normal conditions. (**a**) Dust condition; (**b**) Normal condition.

The platform consists of a face guard mechanism, a shearer drum and ranging-arm mock-up, a 4D millimeter-wave radar, an inclinometer, a data acquisition computer, and backend processing software. The radar records the spatial point cloud and radar return attributes of the target region, whose parameters are shown in Table 1. The inclinometer records the face guard pose for experimental condition identification. The computer stores sensor data and executes point cloud preprocessing, segmentation, bounding-box generation, and distance calculation. Normal and dust conditions were also arranged to test the suitability of radar-based sensing when optical visibility is poor (Figure 2).

### 2.2. 4D Radar Point Cloud Dataset Construction

Raw radar measurements were recorded as ROS bag data. The data extraction workflow reads the radar topic, parses PointCloud2 messages, and exports frame-level point cloud data. Each point retains the spatial coordinates and radar-specific attributes:

(1) $$p_{i}=\left[x_{i},y_{i},z_{i},\rho_{i},a_{i},v_{i}\right],$$

where $x_{i}$, $y_{i}$, and $z_{i}$ are the 3D coordinates in the radar or unified coordinate frame; $\rho_{i}$ denotes radar cross-section (RCS) or a related reflection attribute; $a_{i}$ denotes echo intensity; and $v_{i}$ denotes Doppler radial velocity. The exported point cloud frames were imported into CloudCompare for manual point-level annotation. Points belonging to the face guard were assigned label 1, and background, noise, or unrelated structure points were assigned label 0:

(2) $$y_{i}=\left\{\begin{array}{cc} 1, & p_{i}\in \mathrm{face}\;\mathrm{guard},\\ 0, & p_{i}\in \mathrm{background}/\mathrm{noise}. \end{array}\right.$$

The labeled learning dataset contained 2160 point cloud frames, split into training, validation, and test sets (Table 2). It is worth noting that the 2160 frames were initially selected randomly from the nearly 8000 frames of samples. At the same time, we first randomly divided these frames into six subsets on average and then selected different subsets in a combinatorial manner as the validation set and test set, while the remaining four subsets were used as the training set. Subsequently, we conducted $C_{6}^{1}C_{5}^{1}=30$ experiments and took the average result of these 30 experiments as the final statistical result. All subsequent test data were obtained using the above method. Furthermore, it must be acknowledged that although the above-mentioned K-Fold Cross-Validation method can to some extent ensure the validity of the statistical results and reduce the workload of evaluation, it still has certain limitations. This study will continue to promote the engineering practice verification of the proposed method [19,20].

### 2.3. Point Cloud Preprocessing

The raw radar point cloud contains irrelevant background points and isolated multipath/noise points. A joint filtering strategy was used before model inference. Passthrough filtering first restricts the point cloud to a task-relevant region of interest:

(3) $$P_{\mathrm{roi}}=\left\{p_{i}\in P|x_{\min}\leq x_{i}\leq x_{\max},\;y_{\min}\leq y_{i}\leq y_{\max},\;z_{\min}\leq z_{i}\leq z_{\max}\right\}.$$

Radius filtering then removes local isolated points. For point $p_{i}$, its neighborhood in radius *r* is

(4) $$N_{r}\left(p_{i}\right)=\{p_{j}\in P_{\mathrm{roi}}|∥x_{j}-x_{i}{∥}_{2}\leq r\},$$

where $x_{i}=\left[x_{i},y_{i},z_{i}\right]$. The point is retained if $|N_{r}\left(p_{i}\right)|\geq K_{r}$. In the graduation-design experiment, the representative radius-filter setting was $K_{r}=4$ and r=0.25 m. This joint strategy retains target-region points while reducing distant structural points and local isolated noise. Temporal and spatial fusion were also studied to improve target-region density, but the journal manuscript emphasizes the joint passthrough-radius preprocessing because it gives a clear reproducible input to the segmentation model.

### 2.4. PointNet++ Segmentation with Radar Feature Augmentation

**Figure 3:**
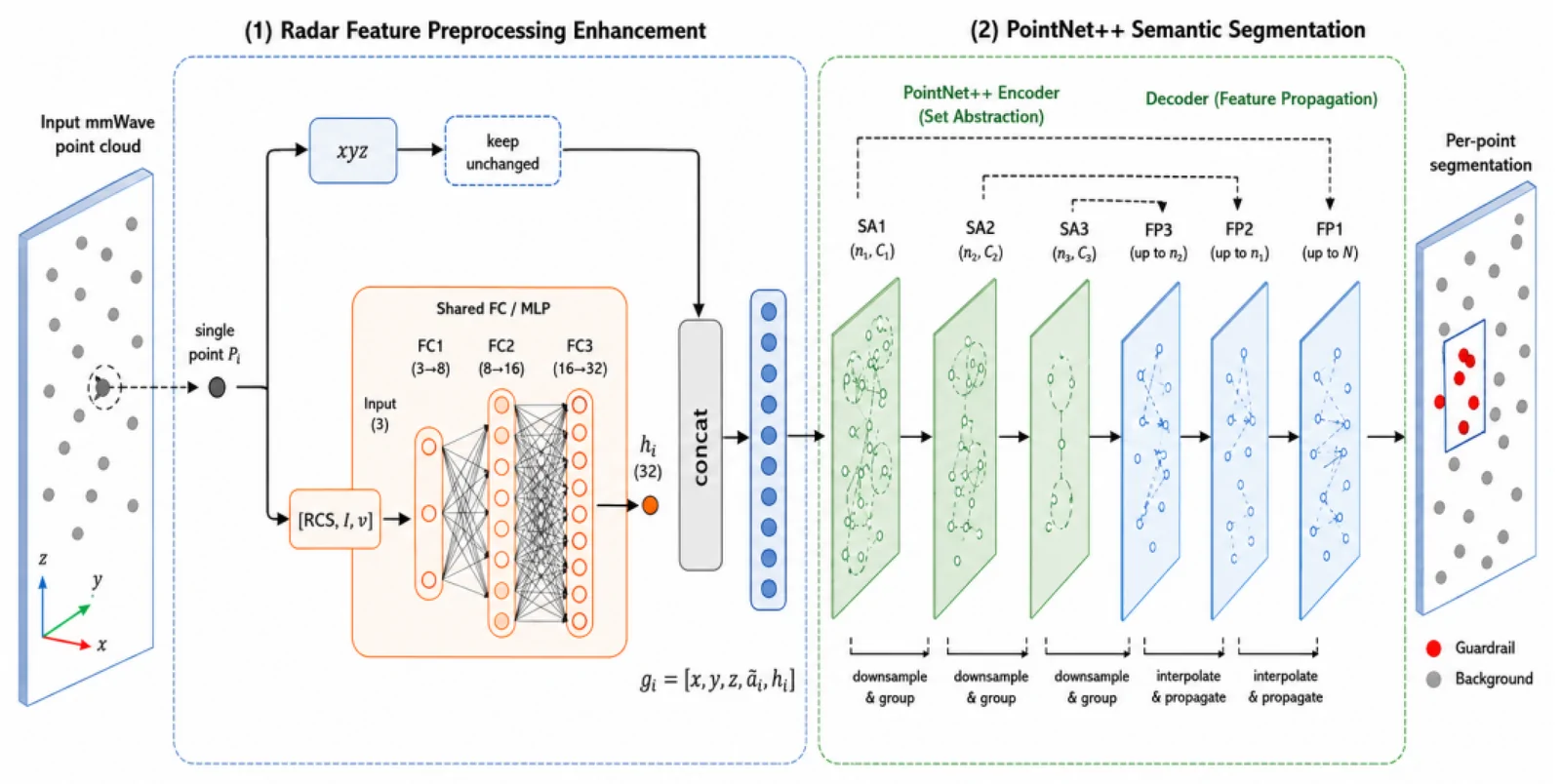
Modified PointNet++ architecture with radar feature preprocessing enhancement. The spatial coordinates are kept unchanged for neighborhood construction, while RCS, intensity, and velocity are passed through a shared multilayer perceptron to form an enhanced point-level radar feature representation.

The segmentation task assigns each point to the background or face guard class. PointNet++ (Figure 3) was selected because it builds local neighborhoods in metric space and learns hierarchical features from unordered point clouds [13]. This is suitable for 4D radar data, where the target is sparse and boundary points are discontinuous.

The raw input channel dimension is 6, corresponding to (x,y,z,RCS,Intensity,Velocity). The coordinates are kept at their physical scale for sampling and grouping. The radar attributes are standardized using training-set statistics:

(5) $${\hat{f}}_{i,d}=\frac{f_{i,d}-\mu_{d}}{\sigma_{d}+\epsilon },$$

where $f_{i,d}$ is the *d*th radar attribute, $\mu_{d}$ and $\sigma_{d}$ are the training-set mean and standard deviation, and ϵ is a small constant. A shared multilayer perceptron maps the standardized radar attributes to a higher-dimensional feature:

(6) $$h_{i}=\phi \left({\hat{\rho }}_{i},{\hat{a}}_{i},{\hat{v}}_{i};\Theta \right),$$

where ϕ is implemented using shared fully connected layers with nonlinear activation. In the designed model, the internal radar feature branch maps three raw radar attributes through 8, 16, and 32 dimensions. The final point representation concatenates the original coordinates, standardized radar attributes, and enhanced radar feature:

(7) $$g_{i}=\left[x_{i},y_{i},z_{i},{\hat{\rho }}_{i},{\hat{a}}_{i},{\hat{v}}_{i},h_{i}\right].$$

The PointNet++ encoder uses set abstraction layers to sample center points, group local neighborhoods, and extract local features. The decoder uses feature propagation layers to interpolate high-level features back to the original point resolution. The final classifier outputs a two-class probability for each point.

### 2.5. Class-Dependent Anchor-Neighborhood Sampling and Training

The face guard points form a minority class in most radar frames. Pure random sampling may therefore generate samples dominated by background points. To increase the frequency with which the model observes the target region while preserving local context, a class-dependent anchor-neighborhood sampling strategy was used. For each training sample, an anchor class is selected according to a predefined class probability. An anchor point is then sampled from that class, and its nearest points in 3D coordinate space are selected to build a fixed-size local training sample. This approach differs from duplicating target points because it keeps the target-background spatial relationship around the anchor.

The training parameters are summarized in Table 3. Adam optimization was used with an initial learning rate of 0.001 for 100 epochs. Each input sample contained 2560 points, and the batch size was 32. Focal loss was used to reduce the dominance of easy background points:

(8) $$L_{\mathrm{focal}}=-\sum_{i=1}^{N}\alpha_{y_{i}}{\left(1-p_{i,y_{i}}\right)}^{\gamma }\log\left(p_{i,y_{i}}\right),$$

where $p_{i,y_{i}}$ is the predicted probability for the true class of point *i*, $\alpha_{y_{i}}$ is the class weight, and γ is the focusing parameter.

### 2.6. Bounding-Box Fitting and Interference-State Output

After semantic segmentation, the predicted face guard point set in frame *t* is

(9) $$G_{t}=\left\{p_{i}\in P_{t}|{\hat{y}}_{i}=1\right\}.$$

If $G_{t}$ is nonempty, an axis-aligned bounding box (AABB) is fitted by the minimum and maximum coordinate values:

(10) $$B_{t}=\left[x_{\min}^{t},x_{\max}^{t}\right]\times \left[y_{\min}^{t},y_{\max}^{t}\right]\times \left[z_{\min}^{t},z_{\max}^{t}\right].$$

**Figure 4:**
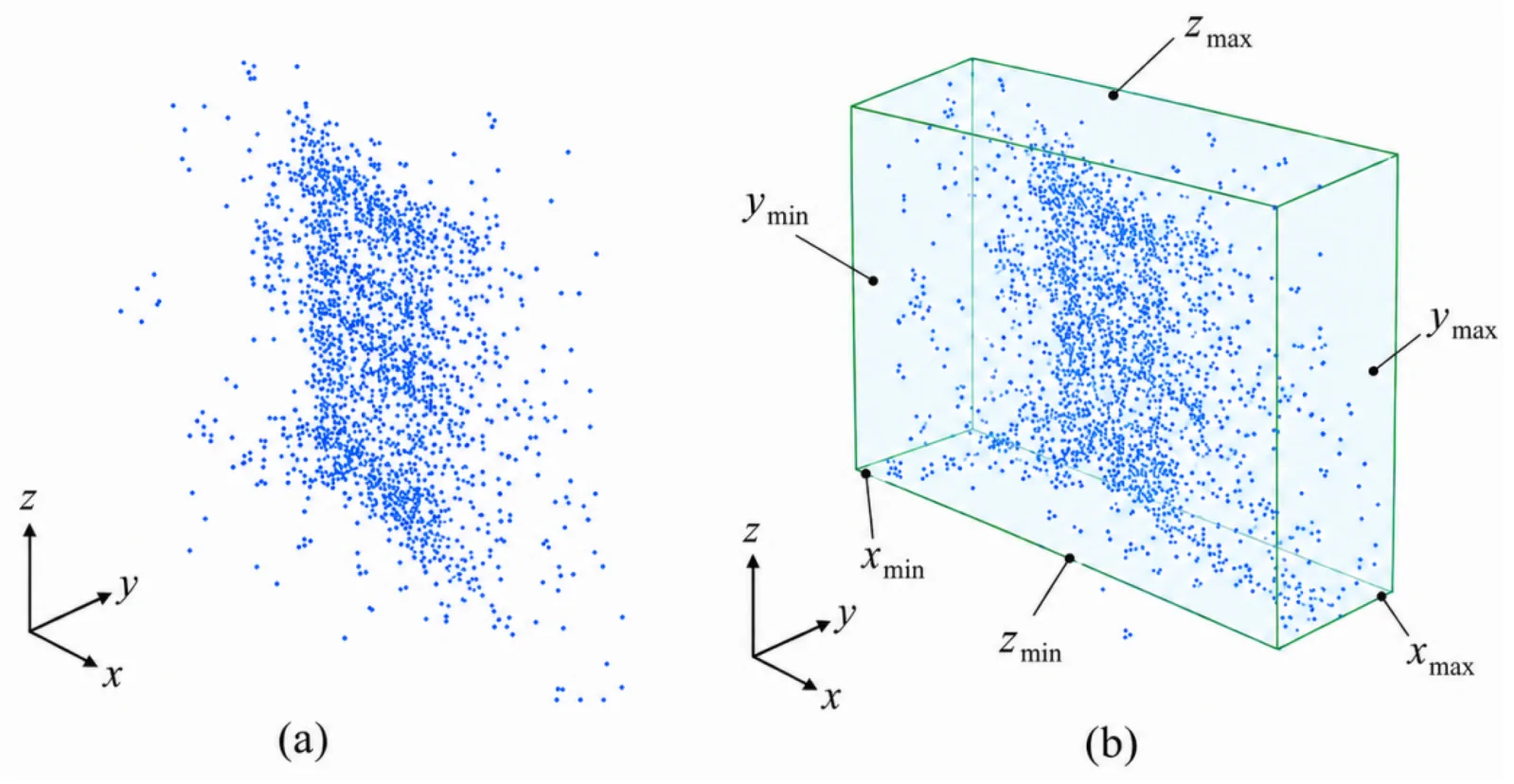
Schematic of axis-aligned 3D bounding-box fitting for segmented face guard radar points. The box is generated from the coordinate extrema of the target point set. (**a**) Collected point cloud; (**b**) 3D bounding box fitting result.

Figure 4 illustrates the bounding-box representation. The AABB is computationally simple and conservative; it does not fully match the face guard surface, but it represents the occupied spatial range required for warning.

The shearer cutting drum and ranging arm are represented by dangerous regions computed from mechanical parameters such as drum radius, drum width, ranging-arm length, and inclination angle. Let $D_{t}$ be the set of representative dangerous points or sampled points on dangerous surfaces in frame *t*. For a point $q=[q_{x},q_{y},q_{z}]$, its distance to $B_{t}$ is

(11) $$d\left(q,B_{t}\right)=\sqrt{\delta_{x}^{2}+\delta_{y}^{2}+\delta_{z}^{2}},$$

with

(12) $$\delta_{x}=\max\left(x_{\min}^{t}-q_{x},0,q_{x}-x_{\max}^{t}\right),\;\delta_{y}=\max\left(y_{\min}^{t}-q_{y},0,q_{y}-y_{\max}^{t}\right),\;\delta_{z}=\max\left(z_{\min}^{t}-q_{z},0,q_{z}-z_{\max}^{t}\right).$$

The frame-level minimum interference distance is

(13) $$d_{t}=\min_{q\in D_{t}}d\left(q,B_{t}\right).$$

Two thresholds define the warning output: $d_{\mathrm{danger}}$ and $d_{\mathrm{warning}}$, where $d_{\mathrm{danger}}<d_{\mathrm{warning}}$. The state is

(14) $$s_{t}=\left\{\begin{array}{cc} \mathrm{Dangerous}, & d_{t}\leq d_{\mathrm{danger}},\\ \mathrm{Warning}, & d_{\mathrm{danger}}<d_{t}\leq d_{\mathrm{warning}},\\ \mathrm{Safe}, & d_{t}>d_{\mathrm{warning}}. \end{array}\right.$$

## 3. Results

### 3.1. Training Convergence and Segmentation Accuracy

The PointNet++ model converged rapidly during the first ten epochs. The training loss decreased from 0.691676 at epoch 1 to 0.134561 at epoch 10. By epoch 10, the recorded IoU, precision, and recall had reached 0.93693, 0.970046, and 0.964846, respectively. After epoch 10, the loss and validation metrics entered a low-amplitude fluctuation range, which is consistent with the sparse and frame-varying nature of radar point clouds. At epoch 100, the final model reached 0.96608 IoU, 0.991587 precision, and 0.974057 recall for the face guard class.

### 3.2. Effect of Point Cloud Preprocessing

Table 4 compares different point cloud preprocessing strategies. Direct use of raw point clouds produced 97.623% precision, 95.961% recall, and 93.679% IoU. Passthrough filtering and radius filtering each improved segmentation performance. The joint passthrough-radius filtering strategy achieved the best result, with 99.126% precision, 97.477% recall, and 96.608% IoU. Relative to raw input, the joint preprocessing improved IoU by 2.929 percentage points.

### 3.3. Effect of Radar Feature Augmentation

Table 5 shows the effect of input feature design. Using only 3D coordinates resulted in 92.235% IoU. Adding raw radar attributes increased IoU to 95.266%, demonstrating that RCS, intensity, and velocity provide useful complementary cues beyond geometry. The radar feature augmentation module further improved IoU to 96.608%, suggesting that nonlinear point-level mapping can better exploit radar return attributes for minority target segmentation.

### 3.4. Component Ablation Study

To evaluate the contribution of the main design choices in the segmentation model, a component ablation study was conducted. The analysis focused on four parts of the proposed network: radar-attribute input, MLP-based radar feature augmentation, class-dependent anchor-neighborhood sampling, and Focal Loss. The full modified PointNet++ model was used as the reference configuration. For each ablation setting, only one component was removed or replaced, while the point cloud preprocessing procedure, 2160-frame learning dataset, partitioning strategy, 100-epoch training schedule, and evaluation metrics were kept unchanged. For consistency among class-level metrics, IoU in this ablation study was calculated from the same positive-class confusion matrix as precision and recall, i.e., IoU=PR/(P+R−PR). In this way, the performance difference between the full model and each ablated model can be used to indicate the contribution of the corresponding component.

As shown in Table 6, using only the 3D coordinates reduced the class-level IoU from 96.608% to 92.235%, indicating that RCS, intensity, and velocity provide effective complementary information for distinguishing the face guard from nearby metal structures and background reflections. When the radar attributes were retained but the MLP-based feature augmentation branch was removed, the IoU decreased to 95.266%. This result suggests that nonlinear mapping of radar-return attributes helps the network extract a more discriminative point-level representation than direct concatenation of raw attributes alone. Replacing class-dependent anchor-neighborhood sampling with random point sampling mainly affected target completeness: recall decreased from 97.477% to 95.692%, and IoU decreased by 2.161 percentage points. This indicates that target-oriented neighborhood construction increases the probability that sparse face guard points are sufficiently represented during training. Replacing Focal Loss with cross-entropy loss reduced recall to 96.184% and IoU to 95.153%, showing that the class-imbalance loss contributes to suppressing the dominance of easy background points. Overall, the four components play complementary roles: radar attributes enrich the input representation, MLP feature augmentation strengthens attribute encoding, class-dependent sampling improves minority-target exposure, and Focal Loss further stabilizes learning under class imbalance.

### 3.5. Qualitative Detection Results

**Figure 5:**
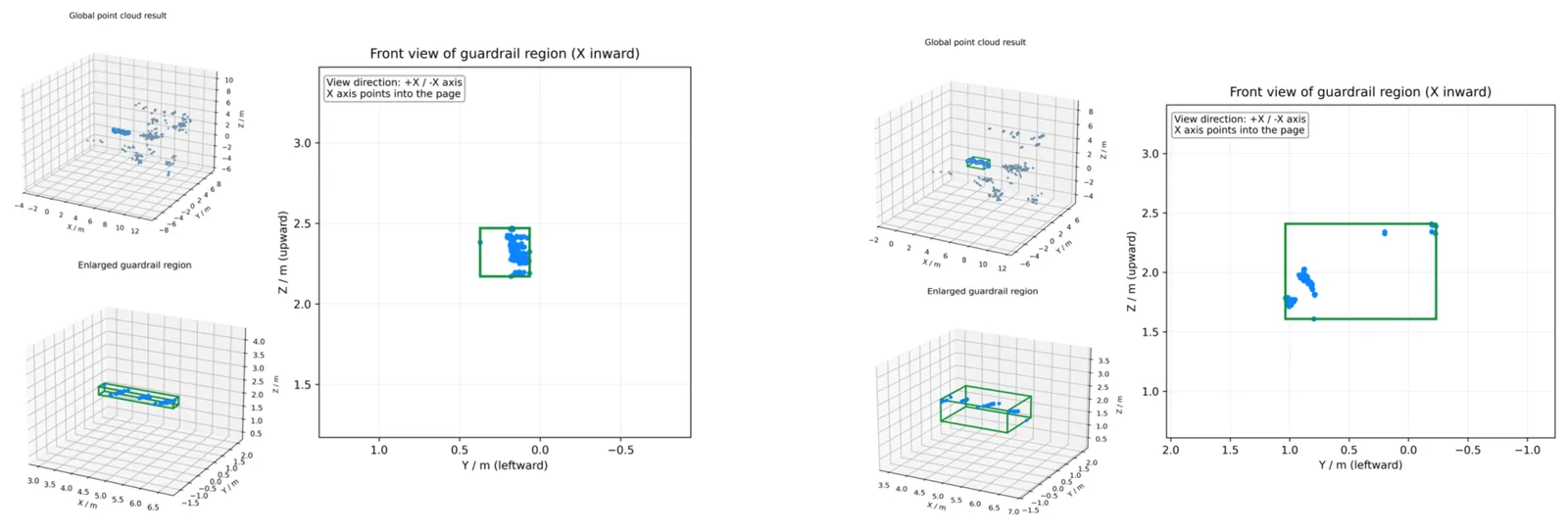
Representative qualitative face guard detection results. (**left**) 0° pose; (**right**) 20° pose. Blue points denote radar point observations and green boxes denote fitted face guard spatial ranges.

Figure 5 shows representative segmentation and bounding-box results for two face guard poses. The model extracts the target points from sparse radar observations, and the fitted bounding boxes cover the major target-point distributions. The qualitative results support the quantitative observation that the proposed model can separate the face guard from surrounding mechanical structures.

### 3.6. Static Face Guard Pose Tests

Static tests were conducted with seven face guard pose angles from 0° to 70°. Table 7 summarizes frame counts, accuracy, recall, mean interference distance, standard deviation, and warning state. Across all pose conditions, the mean accuracy was 98.99%, the mean recall was 97.20%, and all cases were classified as safe. The minimum mean distance among the static tests occurred at 60° (1.3809 m), while the maximum occurred at 0° (1.7097 m). These results indicate that the point-cloud-based bounding-box and distance pipeline can track spatial changes caused by different face guard poses.

### 3.7. Dust-Condition and Dynamic Tests

The dust-condition comparison is summarized in Table 8. Although the dust condition changed the number and distribution of radar points, the pipeline still detected face guard points and produced stable safe-state distance outputs. The mean interference distance differed by 0.048 m between normal and dust conditions.

The dynamic sequence test used 403 frames. To provide a direct comparison with representative point-cloud baselines, PointNet, vanilla PointNet++, and the proposed modified PointNet++ were evaluated on the same dynamic sequence using the same frame-level outputs and downstream distance-calculation procedure (Table 9). PointNet achieved 93.71% accuracy and 82.85% recall, whereas vanilla PointNet++ achieved 95.28% accuracy and 93.04% recall. The proposed modified PointNet++ achieved 99.12% accuracy and 97.39% recall, corresponding to gains of 5.41 and 14.54 percentage points over PointNet, and 3.84 and 4.35 percentage points over vanilla PointNet++, respectively. Its distance standard deviation was 0.2554 m, lower than those of PointNet (0.9265 m) and vanilla PointNet++ (0.3711 m), indicating a more stable frame-wise distance output under this sequence.

## 4. Discussion

The results show that 4D millimeter-wave radar can provide usable 3D observations for face guard detection in a mining-equipment monitoring scenario where optical sensing is vulnerable. The improvement from raw point clouds to joint passthrough-radius filtering indicates that a modest amount of physically motivated preprocessing is beneficial before deep learning. The improvement from coordinate-only input to radar feature augmentation further confirms that radar-specific attributes contribute to target-background discrimination, particularly when the target is sparse and surrounded by other metal structures.

The component ablation results further clarify how the modified PointNet++ model improves face guard segmentation. Radar attributes and the MLP feature branch mainly enhance target-background separability, whereas class-dependent sampling and Focal Loss improve the learning of sparse minority-class target points. These results are consistent with the physical characteristics of 4D radar point clouds, in which the face guard is represented by discontinuous and relatively few points embedded in a metal-rich background.

The proposed method also connects semantic segmentation to safety-state output. This connection is important because point-level recognition alone does not determine whether cutting interference exists. By converting the segmented face guard points into a 3D bounding box and computing the minimum distance from dangerous shearer regions, the method produces a directly interpretable safety variable. The three-level state output is simple enough for integration into a monitoring interface and can be adjusted by selecting application-specific distance thresholds.

Several limitations should be noted. First, the data were collected on an experimental platform, not during full production in an operating coal face. Although dust and pose variation were included, the current experiments do not cover all coupled motions among shearer traction, drum rotation, ranging-arm height adjustment, and hydraulic-support action. Second, the dataset remains relatively small for robust deep-learning generalization. More radar mounting angles, target distances, background structures, dust concentrations, and dynamic machinery interactions should be included. Third, AABB fitting is conservative and efficient, but it may be sensitive to outliers and missing boundary points. Oriented boxes, temporal smoothing, and uncertainty-aware distance estimation could improve stability. Fourth, this draft reports segmentation accuracy and distance outputs but does not yet include end-to-end latency, threshold calibration, or field-failure analysis, all of which are necessary before deployment.

Despite these limitations, the study provides a practical sensing architecture for coal-mining safety monitoring. It demonstrates that 4D radar point clouds can be transformed from sparse measurements into a segmentation-based spatial warning signal. The pipeline also gives a reproducible basis for future work on multi-sensor fusion, temporal tracking, and real-time embedded implementation.

## 5. Conclusions

This paper presents a 4D millimeter-wave radar point cloud sensing pipeline for hydraulic-support face guard detection and shearer cutting-interference warning. The method constructs labeled radar point cloud frames, applies joint passthrough-radius preprocessing, segments face guard points using a modified PointNet++ network, fits a 3D bounding box to the detected target, and calculates the minimum distance to shearer dangerous regions for safety-state classification. On a 2160-frame labeled dataset, the final model achieved 99.126% precision, 97.477% recall, and 96.608% IoU. The component ablation study showed that radar-attribute input, MLP-based feature augmentation, class-dependent sampling, and Focal Loss each contributed to the final segmentation performance; removing these components reduced the class-level IoU by 4.373, 1.342, 2.161, and 1.455 percentage points, respectively. Static tests over seven face guard angles, normal/dust condition tests, and a 403-frame dynamic sequence further verified the feasibility of the pipeline. The results support the use of 4D millimeter-wave radar for active spatial monitoring in visually degraded, metal-rich mining environments, while future work should expand field datasets, improve temporal stability, calibrate warning thresholds, evaluate real-time deployment and impact caused by multipath effect.

## Figures and Tables

**Table 1:** 4D millimeter-wave radar parameters.

Index	Parameter
Model	Oculii Eagle 4D
Operating frequency	77 GHz
Horizontal angle resolution	0.5°
Vertical angular resolution	1°
Field of view	120°×30°

**Table 2:** Dataset splitting for face guard semantic segmentation.

Dataset	Training Frames	Validation Frames	Test Frames	Total Frames
Point cloud frames	1440	360	360	2160

**Table 3:** Hyperparameter configuration for model training.

Parameter	Meaning	Setting
Input features	Point attributes before feature augmentation	(x,y,z,RCS,Intensity,Velocity)
Raw input channels	Number of raw point attributes	6
Output classes	Background and face guard	2
Input points	Points per training sample	2560
Batch size	Samples per update	32
Epochs	Training epochs	100
Optimizer	Parameter update method	Adam
Learning rate	Initial step size	0.001
Loss function	Point-level classification loss	Focal loss

**Table 4:** Face guard segmentation under different point cloud preprocessing strategies.

Point Cloud Input	Precision (%)	Recall (%)	IoU (%)	Interpretation
Raw point cloud	97.623	95.961	93.679	Many background and noisy points retained
Passthrough filtering	98.350	96.858	95.364	Irrelevant spatial regions reduced
Radius filtering	98.503	97.121	95.734	Isolated noise reduced
Passthrough + radius	99.126	97.477	96.608	Target region clearer with fewer outliers

**Table 5:** Face guard segmentation under different input feature configurations.

Input Feature Configuration	Raw Input Dimension	Precision (%)	Recall (%)	IoU (%)
(x,y,z)	3	97.102	94.846	92.235
(x,y,z,RCS, Intensity,Velocity)	6	98.354	96.809	95.266
Radar feature augmentation module	6 raw channels	99.126	97.477	96.608

**Table 6:** Component ablation results of the modified PointNet++ segmentation model.

Ablation Setting	Precision (%)	Recall (%)	IoU (%)	IoU Drop (pp)
Full modified PointNet++	99.126	97.477	96.608	–
Coordinate input only	97.102	94.846	92.235	4.373
Raw radar attributes without MLP augmentation	98.354	96.809	95.266	1.342
Random point sampling	98.641	95.692	94.447	2.161
Cross-entropy loss	98.886	96.184	95.153	1.455

**Table 7:** Static pose test results for different face guard angles.

Face Guard Angle	Frames	Accuracy (%)	Recall (%)	Mean Distance (m)	Std. (m)	State
0°	434	98.82	97.09	1.7097	0.0224	Safe
20°	424	98.76	97.04	1.5225	0.0667	Safe
30°	420	99.08	97.31	1.4721	0.0354	Safe
40°	428	98.91	97.18	1.5106	0.0272	Safe
50°	422	99.24	97.46	1.4766	0.0226	Safe
60°	441	98.73	96.92	1.3809	0.0184	Safe
70°	420	99.37	97.39	1.4033	0.0139	Safe

**Table 8:** Normal and dust-condition test results.

Condition	Frames	Total Points	Detected Face Guard Points	Mean Distance (m)	State
Normal condition	408	3023	177	1.683	Safe
Dust condition	406	3272	149	1.731	Safe

**Table 9:** Dynamic sequence baseline comparison.

Methods	Frames	Accuracy (%)	Recall (%)	Mean Distance (m)	Std. (m)
PointNet	403	93.71	82.85	1.2903	0.9265
Vanilla PointNet++	403	95.28	93.04	1.3698	0.3711
Modified PointNet++ (ours)	403	99.12	97.39	1.3814	0.2554

## Data Availability

The data presented in this study are available on request from the corresponding author.

## References

[1] Man Y. (2019). Research on Monitoring Technology of Cutting Interference between Hydraulic Support Face Guard and Shearer Drum. Master’s Thesis.

[2] Cavieres B., Cruz N., Ruiz-del-Solar J. (2025). Dust Filtering in LiDAR Point Clouds Using Deep Learning for Mining Applications. Sensors.

[3] Li B., Mao S., Zhang H. (2023). Laser Attenuation and Ranging Correction in the Coal Dust Environment Based on Mie Theory and Phase Ranging Principle. Atmosphere.

[4] Parsons T., Seo J., Kim B., Lee H., Kim J.-C., Cha M. (2024). Dust De-Filtering in LiDAR Applications with Conventional and CNN Filtering Methods. IEEE Access.

[5] Chen X., Liu R., Zhang S., Zeng H., Yang X., Deng H. (2020). Research Progress of Underground Millimeter-Wave Radar Point Cloud Imaging and Environmental Map Navigation in Coal Mines. J. China Coal Soc..

[6] Zhang Q., Liu W., Zhang R., Wang Y., Shi M. (2025). Coal Bunker State Monitoring Technology Based on 4D Millimeter-Wave Radar. Coal Sci. Technol..

[7] Liu H., Zhang Z., Chen G., Benndorf J., Yang J. (2025). Automatic Ghost Noise Labeling for 4D mmWave Radar Data in Underground Mine Environments Using LiDAR as Reference. Remote Sens..

[8] Li Y., Shang X. Multipath Ghost Target Identification for Automotive MIMO Radar. Proceedings of the 2022 IEEE 96th Vehicular Technology Conference (VTC2022-Fall).

[9] Ester M., Kriegel H.P., Sander J., Xu X. (1996). A Density-Based Algorithm for Discovering Clusters in Large Spatial Databases with Noise. Proceedings of the Second International Conference on Knowledge Discovery and Data Mining, Portland, OR, USA, 2–4 August 1996.

[10] Duan Z., Shao J., Zhang M., Zhang J., Zhai Z. (2024). A Small-Object-Detection Algorithm Based on LiDAR Point-Cloud Clustering for Autonomous Vehicles. Sensors.

[11] Zhang L., Zhang J., Shi H., Gao L., Hu X., Chen R. Adaptive Point Cloud Clustering Algorithm for Practical Roadside mmWave Radar Systems. Proceedings of the 2024 IEEE 99th Vehicular Technology Conference (VTC2024-Spring).

[12] Qi C.R., Su H., Mo K., Guibas L.J. PointNet: Deep Learning on Point Sets for 3D Classification and Segmentation. Proceedings of the 2017 IEEE Conference on Computer Vision and Pattern Recognition.

[13] Qi C.R., Yi L., Su H., Guibas L.J. (2017). PointNet++: Deep Hierarchical Feature Learning on Point Sets in a Metric Space. Proceedings of the 31st International Conference on Neural Information Processing Systems, Long Beach, CA, USA, 4–9 December 2017.

[14] Cennamo A., Kaestner F., Kummert A. (2021). A Neural Network Based System for Efficient Semantic Segmentation of Radar Point Clouds. Neural Process. Lett..

[15] Shi W., Tong P., Bi X. (2025). Moving-Least-Squares-Enhanced 3D Object Detection for 4D Millimeter-Wave Radar. Remote Sens..

[16] Wu X., Chen Y., Li Z., Hong Z., Hu L. (2024). EFEAR-4D: Ego-Velocity Filtering for Efficient and Accurate 4D Radar Odometry. IEEE Robot. Autom. Lett..

[17] Ding F., Wen X., Zhu Y., Li Y., Lu C.X. (2024). RadarOcc: Robust 3D Occupancy Prediction with 4D Imaging Radar. Proceedings of the 38th International Conference on Neural Information Processing Systems, Vancouver, BC, Canada, 10–15 December 2024.

[18] Jia B., Wei D., Song J., Wang P., Long C. (2024). Interpretability Analysis of 4D Millimeter-Wave Radar for Downhole Applications. Proceedings of the 2024 8th Asian Conference on Artificial Intelligence Technology (ACAIT), Fuzhou, China, 8–10 November 2024.

[19] Wang S. (2026). Automated Fault Detection and Diagnosis of AHUs via Tabular-Based Methods Using Operational Data from a Large Office Building. J. Comput. Civ. Eng..

[20] Wang S. (2026). Class-aware temporal and contextual contrastive framework for semi-supervised automated fault detection and diagnosis in air handling units. Energy Build..

